# Reduced levels of pulmonary surfactant in COVID-19 ARDS

**DOI:** 10.1038/s41598-022-07944-4

**Published:** 2022-03-08

**Authors:** Peter Schousboe, Andreas Ronit, Henning B. Nielsen, Thomas Benfield, Lothar Wiese, Nikolaos Scoutaris, Henrik Verder, Ronan M. G. Berg, Povl Verder, Ronni R. Plovsing

**Affiliations:** 1 Pediatric Research Unit, Department of Pediatrics, Holbaek University Hospital, 4300 Holbaek, Denmark; 2 Department of Infectious Diseases, Hvidovre Hospital, University of Copenhagen, Copenhagen, Denmark; 3 Department of Anesthesia and Intensive Care, Zealand University Hospital Roskilde, Copenhagen, Denmark; 4 Department of Nutrition, Exercise and Sports, Faculty of Science, University of Copenhagen, Copenhagen, Denmark; 5 Department of Infectious Diseases, Zealand University Hospital, Roskilde, Denmark; 6 Department of Biomedical Sciences, Faculty of Health and Medical Sciences, University of Copenhagen, Copenhagen, Denmark; 7 Department of Clinical Physiology, Nuclear Medicine & PET, Rigshospitalet University, Copenhagen, Denmark; 8 Centre for Physical Activity Research, Rigshospitalet, University of Copenhagen, Copenhagen, Denmark; 9 Neurovascular Research Laboratory, Faculty of Life Sciences and Education, University of South Wales, Newport, UK; 10 Department of Anesthesiology and Intensive Care, Hvidovre Hospital, University of Copenhagen, Copenhagen, Denmark; 11 Department of Clinical Medicine, University of Copenhagen, Copenhagen, Denmark

**Keywords:** Biomarkers, Medical research, Pathogenesis

## Abstract

To provide novel data on surfactant levels in adult COVID-19 patients, we collected bronchoalveolar lavage fluid less than 72 h after intubation and used Fourier Transform Infrared Spectroscopy to measure levels of dipalmitoylphosphatidylcholine (DPPC). A total of eleven COVID-19 patients with moderate-to-severe ARDS (CARDS) and 15 healthy controls were included. CARDS patients had lower DPPC levels than healthy controls. Moreover, a principal component analysis was able to separate patient groups into distinguishable subgroups. Our findings indicate markedly impaired pulmonary surfactant levels in COVID-19 patients, justifying further studies and clinical trials of exogenous surfactant.

## Introduction

Like other coronaviruses, severe acute respiratory syndrome coronavirus 2 (SARS-CoV-2) uses the angiotensin-converting enzyme-2 (ACE2) receptor to access and infect pulmonary surfactant producing alveolar type II (ATII) cells^1^. Virus-induced lysis or apoptosis of ATII cells and loss of surfactant in coronavirus disease 2019 (COVID-19)-associated acute respiratory distress syndrome (CARDS) may lead to diffuse alveolar damage, protein leak, and hyaline membrane formation^2,3^.

Pulmonary surfactant consists mainly of dipalmitoylphosphatidylcholine (DPPC) that functions to reduce surface tension, thus stabilizing the alveoli, while increasing pulmonary compliance and reducing the work of breathing^4^. Although exogenous surfactant therapy is effective for premature new-borns with respiratory distress syndrome (RDS), it has failed to improve mortality in non-COVID-19 ARDS^5^. However, CARDS may be associated with an earlier and more profound loss of surfactant, and clinical trials are currently underway to evaluate the effectiveness of exogenous surfactant. Furthermore, exogenous surfactant has recently been reported to improve oxygenation in individual cases of COVID-19 ARDS^6^.

Previously, we have advocated for surfactant deficiency associated with COVID-19^7^. We here, for the first time, show reduced DPPC levels in COVID-19 patients with moderate-to-severe ARDS assessed in bronchoalveolar lavage fluid (BALF) assessed by a recently developed fast POC method.

## Results

All patients had moderate-to-severe impairment of oxygenation at the time of BAL procedure. The overall mortality was high in CARDS (6/11) (Table 1).

**Table 1:** Clinical characteristics of patients with CARDS and non-COVID-19 patients.

ID/patient group	Sex	Age (years)	BMI (kg/m^2^)	PaO2/FiO_2_ (mmHg)^#^	SAPS	Time from intubation to BAL procedure (h)	Time from COVID-19 symptom onset to BAL procedure (days)	Outcome
CO1/Non-COVID-19 ARDS	M	71	28	177	35	39	NA	Survived
CO2/Non-COVID-19 ARDS	M	69	24	115	61	43	NA	Died
CO3/Non-COVID-19 ARDS	F	68	22	175	44	13	NA	Died
CO4/Non-COVID-19 ARDS	M	62	31	113	36	10	NA	Died
V01/CARDS	M	40	21	83	58	65	10	Survived
V02/CARDS	F	65	25	108	72	44	9	Died
V03/CARDS	M	72	33	109	69	20	8	Died
V04/CARDS	M	75	26	70	77	72	20	Survived
V05/CARDS	M	59	22	115	56	41	14	Survived
V06/CARDS	M	75	29	112	54	10	17	Died
V07/CARDS	M	72	23	115	73	15	10	Died
V08/CARDS	M	56	31	123	56	6	12	Survived
V09/CARDS	M	69	25	150	55	12	11	Died
V10/CARDS	M	72	25	125	60	28	9	Died
V11/CARDS	M	67	26	86	59	18	14	Survived

ARDS, acute respiratory distress syndrome; BAL, bronchoalveolar lavage; BMI, body mass index; DXM, dexamethasone; F, female; M, male; PaO2/FiO2, ratio of PaO2 (mmHg) to fractional inspired oxygen; SAPS, simplified acute physiology score.

**Figure 1:**
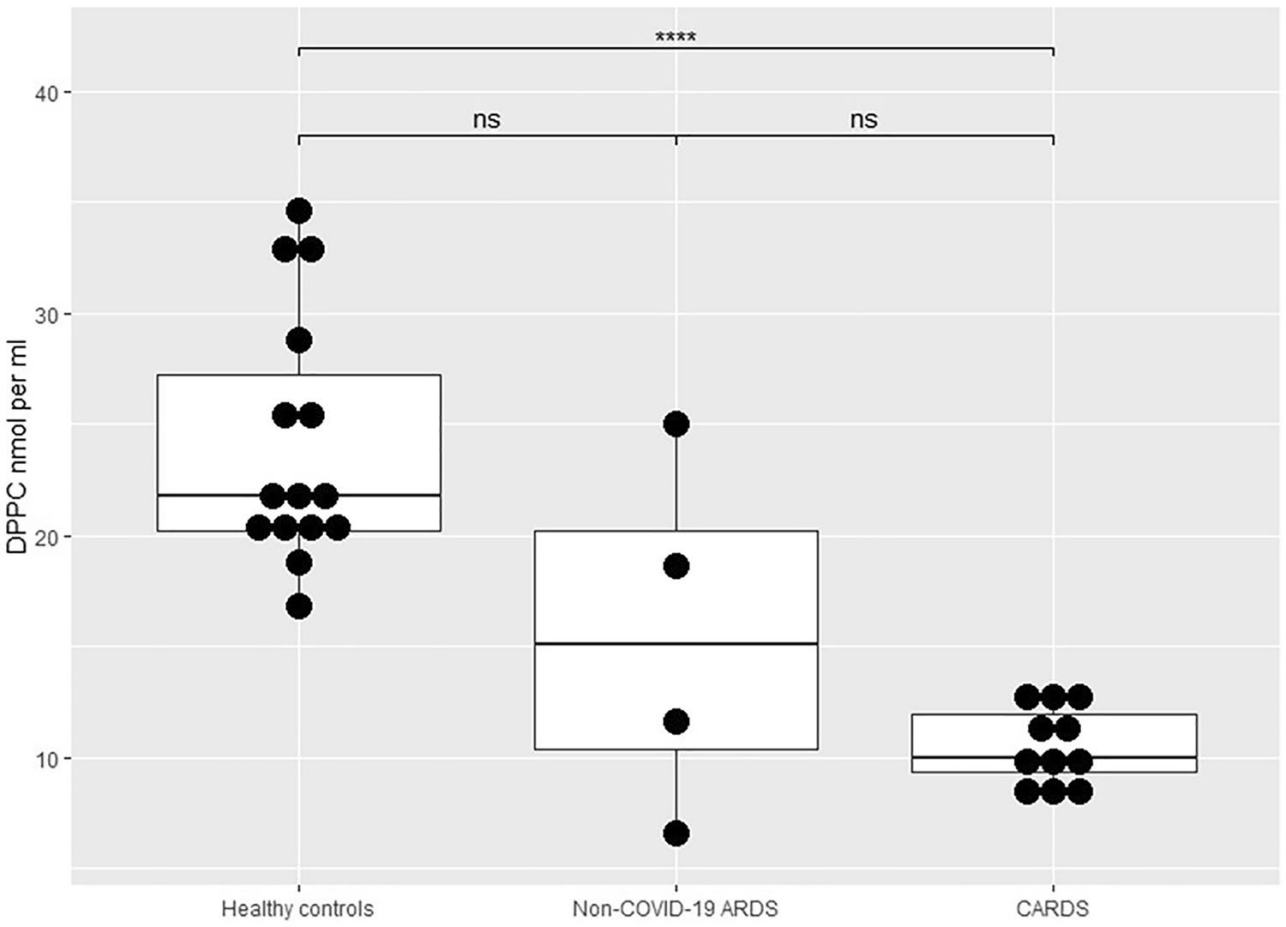
Dipalmitoylphosphatidylcholine (DPPC) levels (nmol/ml) measured in bronchoalveolar lavage fluid (BALF) in 15 healthy controls (M, 15; mean age, 23; SD, 2 years), in four patients with non-COVID-19 ARDS (M/F, 3/1; mean age, 68.5; SD, 3.9 years) and in 11 patients with CARDS (M/F, 10/1; mean age, 65.5; SD, 10.6 years). Boxplots depicts mean values with hinges corresponding to first and third quartile (the 25th and 75th percentiles). Lower and upper whiskers extend to smallest and highest value, respectively. Mann–Whitney *U*-test: *P* < 0.0001. CARDS, COVID-19 associated ARDS; DPPC, dipalmitoylphosphatidylcholine; NS, non-significant. ****P < 0.0001.

**Figure 2:**
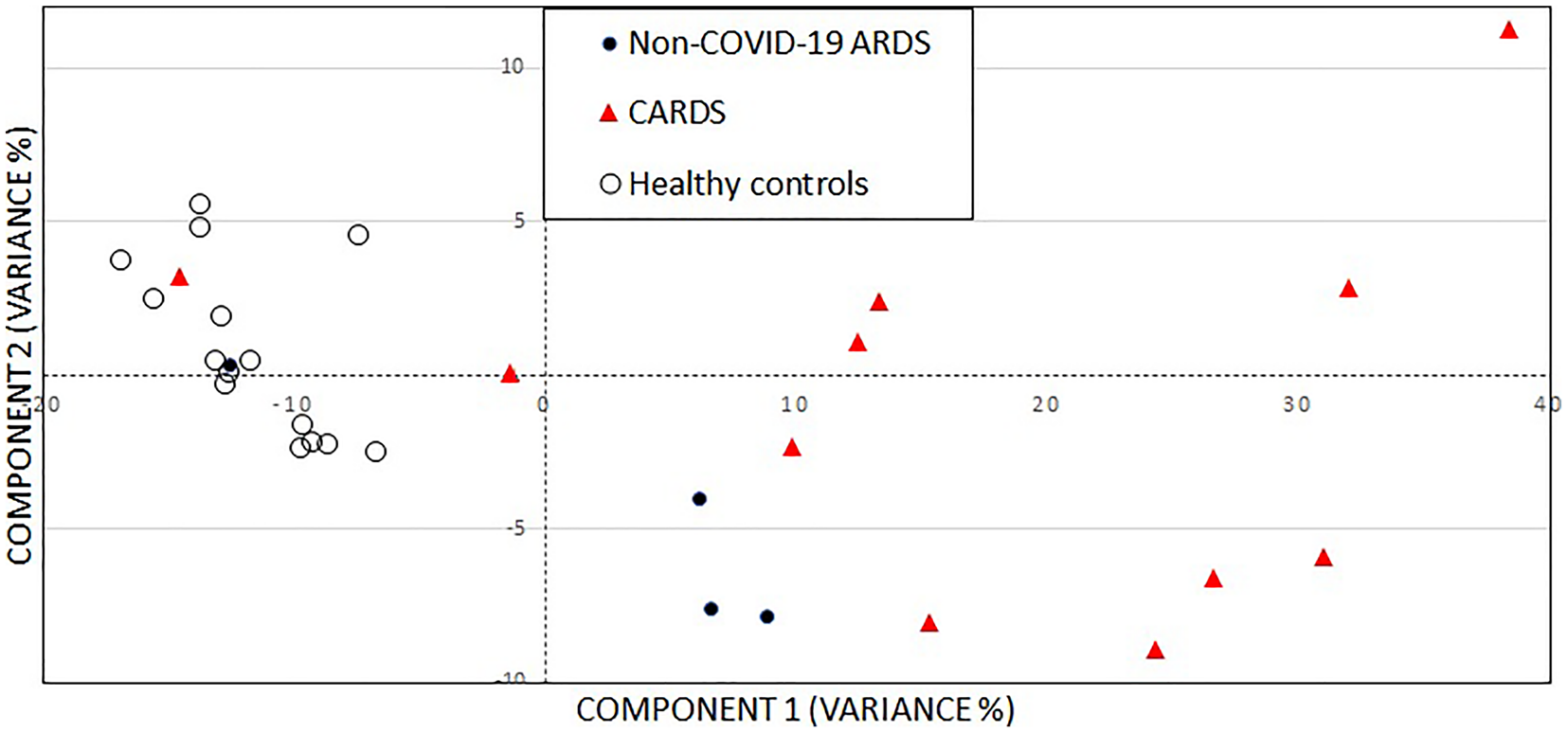
A principal component analysis (PCA) score plot with projection of the data onto the span of the principal components (PC). The spectral matrices were decomposed into two principal components (PCs) by applying singular value decomposition, resulting in a score plot of the first and second PC. Each PC is a linear combination of the wavenumbers of spectra. Before applying PCA, the spectra were baseline corrected (Whittaker smoother) and subsequently normalized by applying standard normal variate methods to avoid intensity variations in the spectra due to the deposition. Two parameters, lambda (λ = 10^5^) and penalty (*P* = 10^–3^) are involved related to the smoothness of the fit and the penalty imposed to the points giving positive residuals in the fit.

DPPC values were different across the three groups (Fig. 1, *P* < 0.0001) with approximately 60% lower levels in CARDS than in HCs. In non-COVID-19 ARDS, DPPC tended to be lower than in HCs (*P* = 0.051) but did not differ from CARDS (*P* = 0.327). A PCA model based on normalized baseline-corrected spectral data is shown in Fig. 2. The principal components separated the data into several clusters with a clear, visual separation of HCs, CARDS and ARDS. The more scattered and heterogenous CARDS and ARDS groups indicated a clear spectroscopic difference between HC and the two groups. The model captured 86% of the total variance, and HC spectra in general were much less scattered due to a lower variability.

## Methods

The study was approved by the Regional Ethics Committee of Copenhagen (approvals H20023159 and H22009131) and registered at ClinicalTrials.gov (NCT04354584). A total of 11 CARDS patients were included in the present study. Data on compartmental immunophenotyping on four patients recruited during the first wave have previously been reported elsewhere^2^. Furthermore, four non-COVID-19 patients with moderate-to-severe ARDS and 15 healthy controls from a previous study were included for comparison^8^. Since all patients were incompetent, informed consent was obtained from next of kin, and the BAL procedure was performed within less than 72 h of mechanical ventilation.

The BAL procedure was performed as previously described^2^. Three successive 50-ml aliquots of isotonic saline were instilled in the medial segment of the right middle lobe and aspirated with low negative suction pressure (< 100 cm H_2_O). Pooled BAL fluid was spun and the acellular supernatant frozen at − 80 °C.

DPPC in BALF was measured by a fast point-of care (POC) method able to give answers within 12 min^9^. In brief, the analysis of thawed acellular BALF (130 ul) was performed by dry transmission Fourier Transform Infrared (FTIR) Spectroscopy. Spectra were obtained by a Sime Diagnostics Alpha + (London, UK) device equipped with a Perkin Elmer SP-2 spectrometer (20 scans; resolution: 4 cm^−1^; aperture: 10 mm). Lamellar bodies were spun down at 4000×g and the precipitate was transferred to a CaF_2_ disk, dried, and a spectrum was obtained from which the concentration of DPPC was derived by an algorithm.

The mathematical algorithm, predicting DPPC levels from the FTIR spectra, was constructed from surfactant reference samples previously assessed by mass spectrometry^9^, and normalized baseline-corrected spectral data were analysed by principal component analysis (PCA). DPPC values between the three groups were compared using non-parametric Kruskal Wallis test, whereas a Mann–Whitney *U*-test was used to compare two groups. Algorithm development and statistical analysis were performed in R software, version 4.0.3/4.0.5. All methods were carried out in accordance with relevant guidelines and regulations.

### Ethics approval and consent to participate

The study was approved by the Regional Ethics Committee of Copenhagen (approvals H20023159 and H22009131) and registered at ClinicalTrials.gov (NCT04354584). Consent was obtained from all enrolled persons either by the persons themselves or by next of kin.

## Discussion

It has been widely speculated that infection of ATII cells may be a driver of CARDS by leading to a profound loss of pulmonary surfactant. Only a handful of studies have investigated local alveolar content, and our data extend on these findings by showing a ~ 60% reduction in pulmonary surfactant in the most severe cases of COVID-19. The POC method has provided us with a tool, capable of measuring the concentration of pulmonary surfactant and with the potential to identify ARDS patients with low surfactant concentration.

Even though previous studies on the use of exogenous surfactant in non-COVID ARDS were unsuccessful, there are several specific considerations supporting its use in COVID-19, i.e. impairment of production and/or reabsorption of surfactant due to ATII cell lysis or apoptosis, altered gene expression patterns with evidence for downregulation of surfactant proteins^10^ or a combination of several factors. It has been speculated that increased alveolar surface tension due to loss of surfactant may predispose to pulmonary barotrauma, and our findings thus provide a potential mechanism of the higher incidence of subcutaneous emphysema, pneumothorax, and pneumomediastinum in mechanically ventilated CARDS patients (~ 17%) compared to non-COVID ARDS (~ 5–11%)^11^. Furthermore, case series of CARDS patients have reported improvement in oxygenation and lung compliance following exogenous surfactant although repeated doses are probably needed given that ATII cell destruction may predominate in CARDS. Taken together, administration of surfactant in CARDS awaits further justification in large clinical trials.

Our findings indicate that surfactant is low in the early progressive phase of CARDS. Accordingly, results from previous non-COVID-19 ARDS studies suggest that earlier administration of surfactant may provide a potential benefit^12^, and POC analysis of tracheal secretes^9^ could potentially be used to estimate pulmonary surfactant levels and identify candidates for exogenous surfactant at earlier timepoints during COVID-19 pneumonia.

Our cross-sectional study shows a significant reduction in DPPC for the CARD patients reflecting disease severity. Despite our limited sample number, a Markov chain Monte Carlo simulation with the application of Bayes theorem showed that the simulated data reflect the raw data. However, larger and ideally well-matched studies should provide more detailed comparisons of surfactant levels according to ARDS etiology.

## Conclusions

CARDS is associated with reduced pulmonary surfactant levels which may severely impose alveolar collapse, impair gas exchange, and increase work of breathing. These changes could also predispose to barotrauma (e.g., pneumothorax), which has increasingly been reported in larger cohorts of CARDS. The POC method for surfactant measurements opens for future clinical studies providing further insight into the therapeutic potential of exogenous surfactant in both CARDS and non-COVID-19 associated ARDS.

## Data Availability

All data generated or analyzed during this study are included in this published article.
